# Changes in Honeybee Behavior Parameters under the Influence of the E-Field at 50 Hz and Variable Intensity

**DOI:** 10.3390/ani11020247

**Published:** 2021-01-20

**Authors:** Paweł Migdał, Agnieszka Murawska, Paweł Bieńkowski, Ewelina Berbeć, Adam Roman

**Affiliations:** 1 Department of Environment Hygiene and Animal Welfare, Wroclaw University of Environmental and Life Sciences, 25 C.K. Norwida St., 51-630 Wroclaw, Poland; agnieszka.murawska@upwr.edu.pl (A.M.); ewelanina.nina@gmail.com (E.B.); adam.roman@upwr.edu.pl (A.R.); 2 Telecommunications and Teleinformatics Department, Wroclaw University of Science and Technology, 27 Wybrzeze, Wyspianskiego St., 50-370 Wroclaw, Poland; pawel.bienkowski@pwr.edu.pl

**Keywords:** E-field, honeybee, worker bee behavior

## Abstract

**Simple Summary:**

A dynamically developing civilization is constantly increasing the demand for electricity. Increasing energy consumption and communication range contributes to the use of different frequencies of the electromagnetic field (EMF). As a result, the natural environment is tightly filled with EMF of various frequencies and intensities. This leaves the entire environment under its influence. The honeybee, as one of the most important pollinators, is constantly influenced by these factors. Studying the influence of this factor on the behavior of the honeybee will help to understand whether these changes pose a threat to this valuable pollinator. Our research showed changes in the behavior of bees under the influence of a 50 Hz E-field. The demonstrated behavioral disturbances may reduce the efficiency of bees as pollinators, which will translate into a decrease in the efficiency of crop production.

**Abstract:**

EM-fields come from both natural and anthropogenic sources. This study aimed to investigate changes in honeybee behavior parameters under the influence of an electric field at 50 Hz and variable intensity. Bees were exposed for 1 h, 3 h, or 6 h to the following artificial E-field intensities: 5.0 kV/m, 11.5 kV/m, 23.0 kV/m, or 34.5 kV/m. Bees in the control group were under the influence of an E-field <2.0 kV/m. Six basic behaviors were selected for bee observation (walking, grooming, flight, stillness, contact between individuals, and wing movement). Our research shows the impact of bee exposure time on behavioral change within groups. Exposure for 3 h caused a decrease in the time that bees spent on behaviors and in the number of occurrences. After 6 h, the parameters increased within the groups, as was the case with 1 h exposure. This may indicate that there is a behavioral barrier that allows the pattern to normalize for some time.

## 1. Introduction

EM-fields represent basic interactions occurring in nature. This phenomenon occurs between particles with electric charge and arises as a result of the combination of an electric and magnetic field. The magnetic field creates a magnetosphere around the Earth that deflects the flux of charged particles (solar wind). It is similar to a magnetic dipole field with geomagnetic poles near the northern and southern poles [1]. The natural electric field of the Earth has its source in a radioactive element, i.e., cosmic rays; thus, it contributes to air ionization. The electricity of the Earth’s atmosphere also includes lightning discharges, electric charges of clouds, and precipitation [2]. The impact of these fields creates a specific electromagnetic field that fills the natural environment. Thus, all living organisms are exposed to its effects. The source of the environmental EM-field is, among others, the Sun, which generates wide-range electromagnetic waves. In addition to natural sources, the electromagnetic field comes as a result of human activity. The rapid development of technology has increased the share of EM-fields from artificial sources. Electromagnetic fields are emitted, for example, by high-voltage lines, antenna radiation, and wireless power transfer [3,4]. The effects of the EM-field can be divided into thermal and nonthermal. Increasing the cell temperature exposes it to major changes. An EM-field with a frequency of 50 Hz may contribute to an increase in the likelihood of many diseases [5]. This frequency is also the most commonly used. The biological effect is achieved by the impact of an EM-field on the body, which results in a physical, biochemical, or behavioral reaction. If these reactions occur within homeostasis, there is a possibility of their reversal [6]. The EM-field affects both vertebrate and invertebrate animals, including insects. Fruit flies (*Drosophila melanogaster* L.) were exposed to a magnetic and electromagnetic field. In the case of the magnetic field, the change was observed at DNA level, mainly manifested by different sensitivity of the genes responsible for the development of various parts of the body and the proliferation of cells. An electromagnetic field at low frequencies had a potentially positive effect on the fruit fly’s organism without causing negative changes in embryonic development, whereas higher frequencies (MHz) reduced reproductive capacity by as much as 60%, along with changes in the rate of cell proliferation and protein synthesis [7]. In the study of Migdal et al. [8], an EM-field at 50 Hz after 1, 3, or 6 h of exposure increased the activities of antioxidant and proteolytic systems of the honeybee in laboratory conditions. Moreover, 12 h of exposition showed changes in the activity of SOD (superoxide dismutase), CAT (catalases), and FRAP (ferric reducing antioxidant power) in all tested groups [9]. Electromagnetic fields (EMFs) can also affect insect behavior. Radiofrequency fields in the MHz range disrupt insect orientation [10]. Extremely low-frequency EMF exposure had effects on the behavior, physiology, and protein expression of desert locusts (*Schistocerca gregaria* F.) [11]. Electromagnetic field exposure for 24 h, 72 h, and 7 days (50 Hz) impaired the latency to escape from noxious heat in American cockroach (*Periplaneta americana* L.) [12].

Studying the E-field’s impact on bees is important because bees, as a flying insect, can approach transmission lines and be exposed to fields with intensity exceeding 10 kV/m; it is even possible to exceed values above 15 kV/m [13]. Honeybee workers fly relatively close to the ground (about 2–3 m above the surface) in free space; when obstacles appear, they fly on average about 5 m above the ground. The flight altitude depends on many factors, including obstacles such as trees and buildings [14]. The purpose of field strength used in the experiment was to analyze the linearity of observed phenomena as a function of the field strength. Bees spend different times obtaining food in the environment, which also affects the time of exposure to the electromagnetic field. Collectors fly for nectar, pollen, and water, covering long distances. Duration depends on many factors; on average, it can be assumed that 1 h is the average time that worker bees spend in the environment in search of food during a single flight, 3 h is the time spent by the worker bee on bringing large and heavy portions of food from distant sources during a single flight, and 6 h is the maximum average time that a worker bee can devote to searching for and obtaining food during a single flight [15]. The honeybee in 2019, during a meeting of scientists from the Royal Geographical Society of London with the Earthwatch Institute, was recognized as the most important organism on earth. Their protection is crucial for the development of civilization. This is becoming more important given the fact that the honeybee (*Apis mellifera* L.) has high economic importance.

## 2. Materials and Methods

### 2.1. Test Organisms

Twenty inseminated queens, originated from the same mother-queen and inseminated with the semen of drones from the same father-queen colony, were individually introduced into 20 queenless colonies. After checking egg-laying, 10 mothers were randomly selected and kept in isolators with empty Dadant combs. Each mother was kept in one insulator (*n* = 10, 435 × 300 mm). Isolators with the bee frames were placed in a bee family of *Apis mellifera carnica* (Pollmann, 1879), in which bee mothers were placed for 12 h. After 24 h of egg-laying, the queens were released, and isolators containing combs with eggs were left within the colonies for further worker-brood rearing. On the 19th day of apian development, the combs with the already sealed worker brood were transferred to the incubator (temperature of 34.4 °C ± 0.5 °C and relative humidity of 70% ± 5%) in which they were maintained within individual chambers for 1 day old bees workers to emerge. The research material consisted of 2 day old honeybee workers.

### 2.2. Experimental Setup

One day old bee workers were placed in wooden cages (20 × 15 × 7 cm), each containing 90 workers and two inner feeders with sucrose solution at a concentration of 1 mol/dm^3^ ad libitum. Each group consisted of 10 cages. Bees in the experimental groups were exposed to the following 50 Hz E-field intensities: 5.0 kV/m, 11.5 kV/m, 23.0 kV/m, and 34.5 kV/m. The control groups were not treated with the artificial E-field; they were under the influence of an E-field <2.0 kV/m. The group name is the combination of E-field intensity and exposure time. For example, bees exposed to a 34.5 kV/m E-field for 6 h had the group name 34.5 kV/m 6 h. The control group was marked with the letter C. The method used was according to Migdał et al. [8].

### 2.3. E-Field Exposure

A homogeneous 50 Hz E-field was generated in the exposure system in the form of a plate capacitor, as in the method of Migdał et al. [8]. The field intensity was fixed to 5.0 kV/m, 11.5 kV/m, 23.0 kV/m, and 34.5 kV/m. The time of exposition was 1, 3, or 6 h. Changes in the homogeneity and stability of E-field intensity were no higher than ±5% in the emitter, to which the bees were exposed during the whole experiment. Field intensity and homogeneity in the test area were verified using the measurements made by an LWiMP-accredited testing laboratory (certification AB-361 of Polish Centre for Accreditation) using ESM-100-m No. 972,153 with calibration certificate LWiMP/W/070/2017 dated on 15 February 2017 issued by the accredited calibration laboratory PCA AP-078. The measurements were done at points of a 10 × 10 × 5 cm^3^ mesh inside an empty emitter (without experimental cages). The stability of the electric field was maintained by permanently monitoring the voltage applied to the exposure system using a control circuit [8].

### 2.4. Behavioral Analysis

Six bees were randomly taken from each cage within the same group, which was placed in a special behavioral assessment station. The total number of worker bees tested in each group was 60. The stand was made of glass, with a height of 20 cm and diameter of 40 cm. Observations of behavior in the program were conducted offline (using recorded material). Recordings of bees lasted 360 s (60 s for adaptation to change in location and 300 s for analysis). Simultaneously, three bees in a container were recorded. After the specified time, bees were exchanged. Bee behavior was recorded using a SONY HDR-CX240E camera (Lund, Sweden). Recorded movies on a memory card were transferred to a computer using the Noldus Observer XT software. Six basic behaviors were selected for observation [16,17]:

- walking—time spent walking,
- grooming—the frequency of cleaning the body surface, tentacles, and tongue,
- flight—between the walls of the container for analysis and between the bottom and the lid,
- stillness—the number of stops where the bee remained stationary for a long time,
- contact between individuals, including trophallaxis and mutual cleansing,
- wings moving—where fanning exposes Nasonov’s gland.

In the first phase, behavioral analysis using the Noldus Observer XT 9.0 software was based on creating the appropriate project in the analyzer environment. The design of the behavioral assessment scheme used a mutually exclusive type of behavior. The project did not use behavior modifiers in the form of changing conditions or interfering with insects, as all individuals were assessed under the same conditions. Independent variables in the form of age, body condition, and damage were also excluded. The possibility of limiting the observations to 300 s (±0.1 s) was used. The average duration of behavior, i.e., how much time the bees from a given group spent on performing this behavior, was analyzed, in addition to the number of occurrences of a given behavior, i.e., how many times during the observation of individuals from the group the selected behavior was observed.

### 2.5. Statistical Analysis

The statistical significance of data within groups and between groups was determined by the Kruskal–Wallis test with Bonferroni correction. For all tests, RStudio [18] was used with a significance level of α = 0.05.

## 3. Results

### 3.1. Control Group

**Figure 1:**
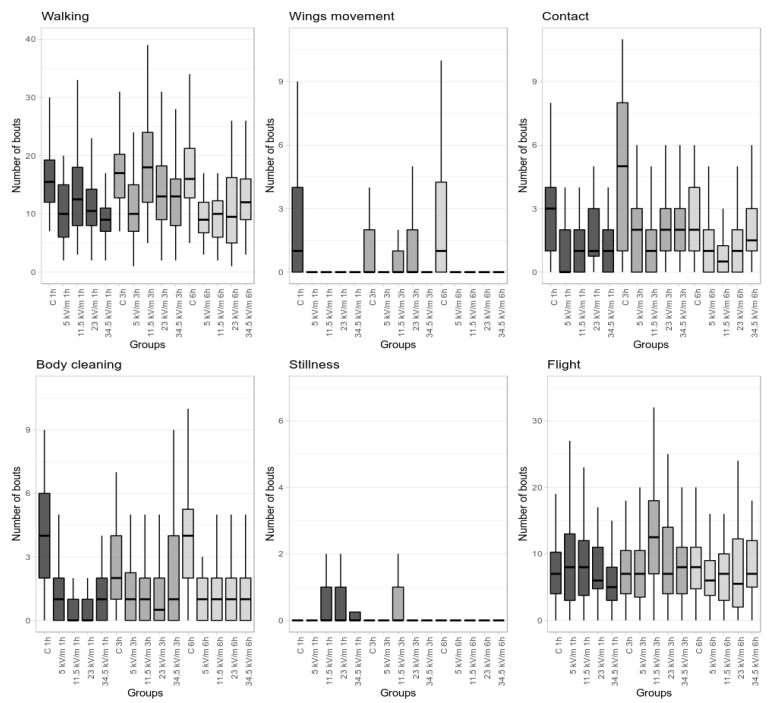
Comparison of the mean number of bouts as a function of exposition time and E-field intensity in and between all groups. The median values are marked by a horizontal line. The lower and upper whiskers denote the minimum and maximum values, respectively.

**Figure 2:**
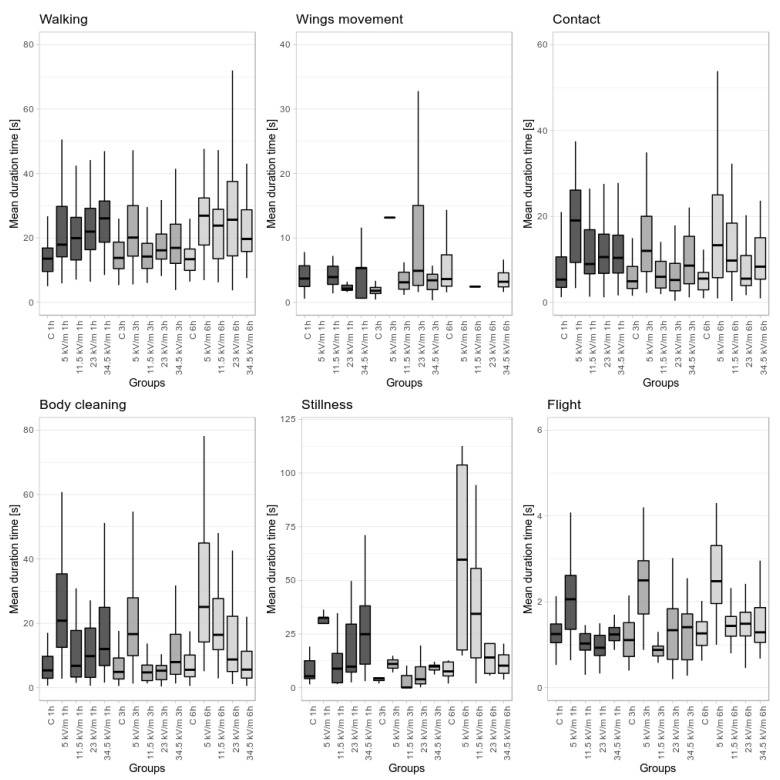
Comparison of the mean duration as a function of exposition time and E-field intensity in and between all groups. The median values are marked as horizontal lines. The lower and upper whiskers denote the minimum and maximum values, respectively.

Worker bees in the control group displayed all types of behavior (Figure 1 and Figure 2, Table 1 and Table 2). There were no significant differences in behavioral patterns between the 1 h, 3 h, and 6 h control groups, except the whole time of stillness, which was longer in the 6 h control group, and the whole time of grooming, where the 6 h control group spent significantly more time on this behavior compared to the 3 h control group.

### 3.2. 5 kV/m Treatment Group

Statistical analysis showed no differences within groups in terms of the number of occurrences and the duration of particular behaviors (see Figure 1 and Figure 2 and Table 1 and Table 2). Regarding the average time spent performing the activity, statistically significant differences were demonstrated for flight, which took more time for bees compared to controls. In the 5.0 kV/m 1 h, 3 h, and 6 h treatment groups, a difference in behavior compared to control and a lack of proportionality were observed. Worker bees in these groups demonstrated longer total flying time compared to the control group. Walking and flying were dominant behaviors. In line with flight time, the average duration was longer for all behaviors. The biggest disproportions were observed in grooming and individual contact behaviors; workers spent 83% and 80.9% more time compared to the control group, respectively. In the 5.0 kV/m 1 h and 6 h treatment groups, wing movement was not observed.

### 3.3. 11.5 kV/m Treatment Group

In the 11.5 kV/m 1 h, 3 h, and 6 h groups, all types of behavior were observed (see Figure 1 and Figure 2 and Table 1 and Table 2). Similarly to previous groups, walking was the most frequent behavior, whereas, in contrast to previous groups, grooming and individual contact were the least frequent behaviors. Walking and flying behaviors demonstrated statistically significant fluctuations. In the 11.5 kV/m 3 h group, the lowest values for grooming time in terms of duration and average time were shown. Compared to controls, bees spent more time on stillness, flying, walking, grooming, and individual contact behaviors and less time on wing movement, which was most frequently observed in this group. Generally, behaviors occurred less frequently but continued ca. 50% longer compared to the control. Bees seemed to be characterized by a greater stability of behavior. With respect to behavioral pattern, the 11.5 kV/m 3 h group was the nearest to the control group. The 11.5 kV/m 1 h and 6 h groups did not demonstrate differentiating values.

### 3.4. 23 kV/m Treatment Group

The 23 kV/m 3 h group demonstrated a statistically significant increase in the number of walking bouts compared to other exposition times (1 and 6 h) (see Figure 1 and Figure 2, Table 1 and Table 2). In relation to the total behavior time, grooming showed a significant decrease in the 23 kV/m 3 h group compared to the 23 kV/m 1 h (37.7% decrease) and 23 kV/m 6 h (59.3% decrease) groups. Wing movement was not observed in the 23 kV/m 6 h group. In the 23 kV/m 3 h group, bees spent significantly more time on this behavior compared to all other groups (Figure 2 and Table 2). Bout duration demonstrated significant fluctuations between groups in relation to most behaviors (walking, grooming, individual contact, and flying) except for stillness, on which bees spent a similar time regardless of the group (Figure 1 and Table 1). In the 23 kV/m treatment group, a disorder in behavioral pattern compared to controls was shown.

### 3.5. 34.5 kV/m Treatment Group

In the number of behavior bouts, there were statistically significant differences among the 1 h, 3 h, and 6 h groups for walking and grooming. The 34.5 kV/m 3 h group showed the highest number of grooming bouts (twofold more than the 1 h and 6 h groups and similar to the control) and the highest number of walking and wing movement bouts (Figure 1 and Table 1). Moreover, the behavior time of wing movement was the longest in this group (Figure 2 and Table 2).

### 3.6. Comparison of All Groups 

Within all behaviors, only stillness did not show significant differences in the number of bouts among all groups.

## 4. Discussion

In this study, the impact of E-fields on honeybees was examined. Only a few publications evaluated this issue; thus, a comparison of the results to other species and factors (pesticides, climate change, temperature, etc.) was necessary.

### 4.1. Control Group

The bees used in this study (at age 2 days ± 6 h) should exhibit all examined types of behavior, including flying, which, according to Vance et al. [19], are not dependent on age. Actually, they were all observed in the control group; however, the flying possibilities were changed in the treated groups. Williamson et al. [20], in studying the impact of pesticides on honeybee behavior, observed a similar behavioral pattern in their control group compared to our control group. Results points to the right behavioral pattern under laboratory conditions. According to Shpigler et al. [21], who studied the impact of pesticides on honeybee behavior, there is a high probability of repeating a behavioral pattern under the influence of the same factor. In addition, the repeatability and the proportionality of a behavior strongly depend on the number of equally observed individuals. In our study, only one bee was observed at a time, as, according to Kimura et al. [22], a higher number would disturb the counting possibilities and generate significant error. The activity observed in our study was typical for the temperature, humidity, and season conditions [23].

### 4.2. 5 kV/m Treatment Group

The longer average time of flight could be evidence of the higher activity of bees; however, interpretation is difficult due to a lack of similar studies in the available literature. Nonetheless, one should remember that pesticides damage different systems in the body, e.g., nervous or muscular system. According to Margotta et al. [24], excessive flight activity causes an increase in oxidative system activity, which can lead to a decrease in bee vitality. The differences between behaviors and the lack of proportionality in the number of bouts compared to controls indicate behavioral disorders [20]. The significance of contact between individuals is still under investigation, as it has been proven that an increase in individual contact frequency is a symptom of stress, with a willingness to communicate to obtain information. This also facilitates regulation of the behavior of bees forming the community [25]. Furthermore, it is a significant element in pheromone transmission, allowing bees to evaluate the condition of individuals and modulating their roles [26,27]. Thus, a disorder of this behavior results in a modification of community functioning and of relationships between individuals. The lack of wing movement in the 5.0 kV/m 1 h and 5.0 kV/m 6 h groups is not natural for bees. Honeybees use wing movement to spread the secretions of the Nasonov gland and to maintain the temperature in the hive [28].

### 4.3. 11.5. kV/m Treatment Group

In this group, grooming and individual contact were the least frequent behaviors. The intensity of grooming by eastern honeybees is especially evident when they are in contact with a pathogen, e.g., *Varroa destructor* mite [29]. Carniolan honeybees increase the intensity of this behavior when they come into contact with substances that disturb their perceptual abilities [30]. Grooming behavior is caused by irritation of the sensory hairs located on the body surface and in situations when the chemical receptors are stimulated [31]. A decrease in the frequency and duration of wing movement may indicate dysfunctions in the organs of bees related to movement, which are necessary for the proper functioning of individuals, as presented in studies by Wells et al. [32], who analyzed the correlation between bee infection with *Nosema* spp. spores and wing deformation virus, as well as flight length and activity.

### 4.4. 23 kV/m Treatment Group

A higher number of walking bouts may negatively affect the transfer of information between individuals during waggle dances, in which the speed and direction of movement of the “dancer” bee play an important role [33]. Chaotic information leads to a misdirection of forager bees, which significantly increases the cost of flight and threatens the survival of the bee family [34,35]. The strong decrease in total and average grooming time denotes a serious disorder in the bees’ natural behavior; grooming is essential due to their strong hygiene instincts, and because sticking of the foragers to pollen would make it difficult to return to the nest and receive information [36]. A disturbing stimulus in the form of an E-field may make it difficult for bees to identify specific chemical signals from the atmosphere of the hive, whereby chaos would lead to impaired learning ability and disorganization in bee families and of the community [37].

### 4.5. 34.5 kV/m Treatment Group

The significantly increased walking and grooming activity indicates a desire to escape and the increased motor activity of bees. Similar observations were made by Tosi and Nieh [38], who studied the effect of thiamethoxane on phototaxis in honeybees. They showed a relationship between the length of contact with the pesticide and the increasing frequency of walking speed. When using 1.34 ng of thiamethoxane per bee, increases in the speed (+109%) and movement time (+44%) of bees were observed. Prolonged exposure (over 60 min) caused a decrease in the motion of insects. Opposite results were obtained in studies on thiamethoxane conducted by Aliouane et al. [16], who showed no significant differences in motor function between bees affected by this neonicotinoid and the control group. They used bees at the age of 12 days in the study, which may be the reason for these differences. Walking is an important element when moving to the flower and obtaining nectar; thus, such changes may reduce the usefulness of bees as pollinators [39].

### 4.6. General Comparison among Groups

Changes in the behavior of bees were related to both the duration of exposure and the intensity of the field. In the case of the 5 kV/m 1 h and 6 h groups and 23 kV/m 6 h group, no wing movement was observed in bees. It is difficult to identify the reason due to the shortage of studies in this area. Most often, exposure for 3 h caused a decrease in the time that bees spent on behaviors and the number of occurrences, regardless of the E-field intensity used. After 6 h, the parameters increased within groups, as was the case with 1 h exposure, regardless of the E-field intensity used. This may indicate that there is a behavioral barrier that allows the pattern to normalize for some time (in relation to the control sample). Exceeding this point causes a further decrease in the incidence of a behavior and an increase in the time the insect spends on the behavior. In our research, the tolerance point was 3 h, while 1 h exposure caused changes, which to some extent shows that normalizing the behavioral pattern requires more than 1 h, but less than 3 h, at which point the behavior is less diverse. The time when the behavior was again deregulated was a period of about 6 h. Despite this, no return to reference values (control) was observed in any of the cases. Comparing all groups, the 5 kV/m group had the lowest mean number of behavior occurrences, but the highest mean duration was observed. Similar behavioral disorders were observed in the work of Greenberg et al. [40] on the effect of overhead high-voltage lines on bee colonies. This study showed an increase in motor activity, irregularities in hive propolis, reduced birth growth, and an increase in the percentage of maternal loss relative to the control. In beehive conditions, bees improperly rebuilt nurseries, and the bees were shot dead. Families were also characterized by low winter hardiness. This is similar to the changes seen in the 5 kV/m and 11.5 kV/m groups for all exposure times in this research. Changes in bee behavior can also trigger radiofrequencies, as shown in [41]. Placing two cell phones in the hive increased the volume of sounds in the hive. According to researchers, this may have been due to a disruption in communication among bees. While studying radiofrequencies in field conditions, Sharma and Kumar [42] noticed a decrease in the efficiency of bee colonies and a decrease in reproductive abilities. It was interesting that the frequency of occurrence and the total time that bees spent on wing movement increased for the 11.5 kV/m 3 h and 34.5 kV/m 6 h groups, in contrast to the other parameters, which decreased or remained at a level similar to the control group. Kirschvink et al. [43] suggested, however, that bees require a 100 μT induction level to be able to distinguish between E-fields at 60 Hz, which is at least an order of magnitude higher than static fields used in our research [44]. The same researchers, according to bee waggle dance analysis, stated that the magnetoreceptor system does not work at EMF (electromagnetic field) greater than 500 μT, which also significantly affects the learning and motor skills of bees. Our research shows that the selected frequency, as well as the intensity and duration of exposure, affects the bees’ behavioral patterns. Shepherd et al. [45] confirmed the relationship between low-frequency E-fields and the increased frequency of wing movement during bee flight. Flight is a very energy-consuming motor behavior, which is strongly correlated with bee life expectancy [46]. Therefore, exposure of bees to EMF may result in increased energy demand during flight; thus, during periods of poor use, there may be a situation in which the worker will not be able to return to the hive [47].

## 5. Conclusions

The proper functioning of a bee in the environment whereby it fulfills its role as a pollinator is associated with the correct arrangement of individual behaviors. This is also important when bees make “decisions” and communicate through a waggle dance [33,48]. In light of the current research results, it is difficult to determine how flexible the behavioral patterns of honeybees are and where the limit of their tolerance to environmental impact lies. The intensity and length of exposure have a negative impact on the behavior of honeybee workers. These factors disrupt the natural (defined in the control group) behavioral pattern. The results of our laboratory studies show changes in selected behaviors. The natural consequence of these studies involves similar simulations in natural conditions, which may complete the results and indicate the consequences for bees living in the natural environment.

## Figures and Tables

**Table 1:** Comparison of the mean number of bouts as a function of exposition time and E-field intensity in and between all groups.

Behavior	Groups and Time of Exposition														
	C—Control			5 kV/m			11.5 kV/m			23 kV/m			34.5 kV/m		
	1 h	3 h	6 h	1 h	3 h	6 h	1 h	3 h	6 h	1 h	3 h	6 h	1 h	3 h	6 h
Walking	16.8 ^ab^	16.9 ^ab^	17.0 ^ab^	11.4 ^cd^	11.9 ^cd^	9.9 ^d^	13.9 ^bc^	19.1 ^a^	11.6 ^cd^	11.7 ^cd^	13.5 ^bc^	11.4 ^cd^	9.7 ^d^	14.4 ^bc^	12.6 ^cd^
Flight	8.0 ^b^	8.9 ^b^	9.8 ^ab^	10.2 ^b^	9.2 ^b^	7.6 ^b^	9.3 ^b^	13.9 ^a^	9.4 ^b^	8.0 ^b^	9.2 ^b^	9.4 ^b^	6.9 ^b^	9.7 ^b^	8.8 ^ab^
Body cleaning	4.4 ^a^	3.1 ^ab^	4.0 ^a^	2.0 ^cd^	2.2 ^bcd^	1.6 ^cd^	1.8 ^d^	3.1 ^bcd^	2.0 ^cd^	1.7 ^d^	2.3 ^cd^	1.9 ^cd^	1.7 ^cd^	3.6 ^bc^	2.2 ^cd^
Contact between individuals	4.2 ^ab^	5.6 ^a^	2.8 ^abc^	1.9 ^e^	2.7 ^bcde^	1.9 ^de^	1.9 ^de^	2.3 ^cde^	1.8 ^e^	2.4 ^bcde^	2.8 ^bcd^	2.3 ^cde^	2.0 ^cde^	2.8 ^abc^	2.3 ^bcd^
Wing movement	4.1 ^a^	3.0 ^b^	3.9 ^a^	NO	1.0 ^d^	NO	1.0 ^cd^	6.6 ^bc^	1.0 ^d^	1.3 ^d^	3.6 ^bc^	NO	1.2 ^d^	4.3 ^bcd^	2.8 ^cd^
Stillness	1.6 ^abcd^	1.9 ^abcd^	2.1 ^abcd^	1.8 ^cd^	1.0 ^d^	1.0 ^d^	2.3 ^a^	1.6 ^ab^	1.3 ^abcd^	1.4 ^abc^	1.4 ^bcd^	1.2 ^cd^	1.9 ^abcd^	2.0 ^cd^	2.2 ^abcd^
Sample size (*n*)	60			60			60			60			60		

Different letters (a, b, c, d, e) on the chart indicate statistical differences at the level of *p* ≤ 0.05. NO—not observed.

**Table 2:** Comparison of the mean duration as a function of exposition time and E-field intensity in and between all groups.

Behavior	Groups and Time of Exposition														
	C—Control			5 kV/m			11.5 kV/m			23 kV/m			34.5 kV/m		
	1 h	3 h	6 h	1 h	3 h	6 h	1 h	3 h	6 h	1 h	3 h	6 h	1 h	3 h	6 h
Walking	14.2 ^d^	15.5 ^cd^	14.5 ^d^	22.9 ^ab^	22.1 ^ab^	26.2 ^a^	21.8 ^abc^	15.4 ^cd^	24.8 ^ab^	23.6 ^ab^	21.4 ^bcd^	33.8 ^a^	28.0 ^a^	21.7 ^bcd^	22.5 ^ab^
Flight	1.3 ^bcd^	1.2 ^cde^	1.3 ^bcd^	2.1 ^a^	2.5 ^a^	2.8 ^a^	1.3 ^def^	0.9 ^f^	1.5 ^b^	1.2 ^ef^	1.3 ^bcd^	1.5 ^b^	1.3 ^bcd^	1.3 ^bcd^	1.6 ^bc^
Body cleaning	6.4 ^e^	8.9 ^e^	8.5 ^e^	39.5 ^a^	34.2 ^ab^	33.8 ^a^	18.1 ^bcde^	6.0 ^e^	34.7 ^a^	16.8 ^bcde^	6.6 ^e^	30.8 ^abcd^	19.6 ^abc^	11.8 ^cde^	11.6 ^de^
Contact between individuals	6.9 ^de^	7.7 ^de^	6.1 ^e^	36.2 ^a^	14.9 ^a^	17.3 ^abc^	18.9 ^ab^	8.1 ^bcde^	17.0 ^abc^	11.6 ^abc^	10.6 ^cde^	10.1 ^bcde^	18.1 ^abc^	15.7 ^abcd^	12.5 ^abcd^
Wing movement	5.1 ^ab^	2.0 ^ab^	7.2 ^a^	NO	13.2 ^a^	NO	4.1 ^ab^	3.7 ^ab^	2.4 ^ab^	2.3 ^ab^	15.4 ^a^	NO	4.7 ^ab^	3.9 ^ab^	3.9 ^ab^
Stillness	9.9 ^ab^	5.7 ^b^	11.9 ^ab^	28.4 ^a^	11.0 ^ab^	61.7 ^a^	18.3 ^ab^	3.6 ^b^	36.7 ^a^	21.2 ^ab^	20.9 ^ab^	18.9 ^ab^	27.0 ^a^	9.4 ^ab^	11.4 ^ab^
Sample size (*n*)	60			60			60			60			60		

Different letters (a, b, c, d, e, f) on the chart indicate statistical differences at the level of *p* ≤ 0.05. NO—not observed.

## Data Availability

The data presented in this study are available on request from the corresponding author. The data are not publicly available due to the agreement with funding bodies.
